# Non-Pharmacologic Intensive Interdisciplinary Pain Treatment in Pediatrics: Impact on Symptoms, Daily Functioning, and the Family Unit

**DOI:** 10.3390/children11020197

**Published:** 2024-02-04

**Authors:** Jessica Campanile, Becky Wu, Maitry Sonagra, Mackenzie McGill, Daneka Stryker, Jamie Bradford, Jennifer Sherker, Tami Konieczny, David D. Sherry, Sabrina Gmuca

**Affiliations:** 1 Children’s Hospital of Philadelphia, Perelman School of Medicine, University of Pennsylvania, Philadelphia, PA 19104, USA; jessica.campanile@pennmedicine.upenn.edu (J.C.); bradfordj@chop.edu (J.B.); sherry@chop.edu (D.D.S.); 2 Department of Child and Adolescent Psychiatry and Behavioral Sciences, Children’s Hospital of Philadelphia, Philadelphia, PA 19104, USA; wubs@chop.edu; 3 Haddonfield Psychiatry and Therapy, Haddonfield, NJ 08033, USA; 4 Department of Pediatrics, Division of Rheumatology, Children’s Hospital of Philadelphia, Philadelphia, PA 19104, USAmcgillm1@chop.edu (M.M.); 5 Clinical Futures, Children’s Hospital of Philadelphia, Philadelphia, PA 19104, USA; 6 PolicyLab, Children’s Hospital of Philadelphia, Philadelphia, PA 19104, USA; 7 Heart Center, Center for Integrative Brain Research, Seattle Children’s Hospital, Seattle, WA 98105, USA; 8 Drexel University College of Medicine, Drexel University, Philadelphia, PA 19129, USA

**Keywords:** chronic pain, adolescents, psychosocial health, Rheumatology/Musculoskeletal Disorders

## Abstract

**Objectives:** To assess non-pharmacologic treatment outcomes pertaining to health-related quality of life (HRQoL) in youth with chronic idiopathic pain and their families. **Methods:** We conducted a retrospective cohort study of 115 youth with chronic idiopathic pain enrolled in a non-pharmacologic, hospital-based intensive interdisciplinary pain treatment (IIPT) program. HRQoL measures for the patient (Pediatric Quality of Life Inventory [PedsQL] short form) and family unit (PedsQL Family Impact) were collected on admission and discharge as part of routine clinical care. Changes in PedsQL scores were calculated using the Wilcoxon signed-rank test. Multivariable linear regression was used to explore factors associated with patient-level HRQoL. **Results:** Both individuals and the family unit reported that their HRQoL improved in all domains by program completion. Improvements in pain and allodynia were present for program participants at the time of completion as well as at the 3-month follow-up, suggesting durability of these effects. **Conclusions:** A non-pharmacologic IIPT program is a compelling treatment for pediatric and adolescent chronic idiopathic pain, for both patients and the family unit. Patients participating in this program had positive treatment outcomes with significantly improved subjective and objective measures of physical, emotional, social, and cognitive function.

## 1. Introduction

Non-pharmacologic management of pediatric chronic pain has increasing relevance, given the repercussions of the ongoing opioid epidemic. Unmanaged chronic pain in youth has been associated with an increased risk of unresolved pain and opioid use disorder in adulthood [1,2]. Ideal care for young people with chronic pain consists of an interdisciplinary treatment approach based in a biopsychosocial framework [3,4]. Data from the 2007–2015 National Ambulatory Medical Care Survey showed medication as the most prescribed form of treatment, at 59.3% in young people [5]. However, evidence suggests that physical and pharmacologic intervention may have equivocal benefits, and that psychological interventions may be the most durable for decreasing certain symptoms or metrics [6]. This highlights the need for further dissemination of evidence-driven, non-pharmacologic pediatric chronic pain care that incorporates a biopsychosocial model of health.

In a 2022 World Health Organization systematic review of pediatric chronic pain management, comparing psychological, physical, and pharmacologic interventions, each modality showed decreased pain separately, but these effects were not maintained at follow-up visits. Investigating the durability of any of these therapies is paramount to addressing the chronicity of these patients’ pain [6].

Our group previously reported improvements in pain reduction through an interdisciplinary model, consisting of aerobic training, cognitive behavioral therapy, and self-regulation skills [7]. These findings, however, are outdated, having been published in 2015. Furthermore, this study was limited to youth with juvenile fibromyalgia syndrome and did not include a more heterogenous sample of chronic idiopathic pain syndromes. Lastly, this study did not include the family unit’s health-related quality of life (HRQoL) as a treatment outcome.

The purpose of this current study was to assess the physical and psychosocial treatment outcomes and HRQoL at the level of the patient and family unit for youth with chronic idiopathic pain enrolled in intensive interdisciplinary pain treatment (IIPT). Recently, Palermo et al. determined the core clinical outcomes in pediatric chronic pain clinical trials to be pain severity, pain interference with daily living, overall wellbeing, and adverse events [8]. With these outcomes in mind, we evaluated outcomes within medical, socioemotional, and family domains through patient and family HRQoL surveys. Specifically, we aimed to examine changes in patient and family HRQoL scores via participation in the program and to explore the durability of these changes at the time of the first clinic follow-up. We hypothesized the following: (1) HRQoL, at the level of patient and family unit, improves following completion of IIPT, and (2) the pain intensity score, Functional Disability Inventory (FDI) score, and measures of physical therapy (PT) and occupational therapy (OT) functioning all improve upon program completion and will endure post-discharge to the patient’s first follow-up visit (within ~2–3 months of program completion).

## 2. Methods

### 2.1. Study Design and Participants

This was a retrospective cohort study of children and adolescents with idiopathic chronic pain, who were enrolled in a non-pharmacologic, hospital based IIPT program from December 2016 to April 2019 and who completed Quality Improvement (QI) survey measures. Patients and one of their caregivers completed the Pediatric Quality of Life Inventory short form (PedsQL SF-15) questionnaire [9] and family impact module [10] pre- and post- program as a part of their participation in a quality improvement initiative, which served as the main study data. These surveys have demonstrated responsiveness, construct validity, and predictive validity with pediatric patients in the hospital setting, associating lower scores on the PedsQL to align with increased health challenges, such as the presence of multiple complex conditions, longer length of stay in the hospital, higher risk of readmission or return emergency department visits [11]. These pre- and post-program data were further complemented by retrospective chart review and abstraction of all readily accessible and viewable data from the electronic medical record for these patients. The study protocol received an exemption by the Institutional Review Board at Children’s Hospital of Philadelphia (IRB 19-017168).

### 2.2. Intervention

Prior to the interdisciplinary intensive pain rehabilitation program, children and adolescents were first evaluated in the outpatient clinic by providers including a medical provider (physician and/or advance practice provider), psychologist, and physical and occupational therapists. The psychologist determined candidacy for IIPT from a mental health safety standpoint (e.g., suicidality risk, eating disorder, or severe mental health condition requiring stabilization prior to participation in the program). All patients were provided outpatient treatment recommendations, however, patients with persistent pain who required a higher intensity of care, or for whom outpatient treatment had not proven successful, were referred for the IIPT.

The IIPT consisted of two admission types—either day hospital or inpatient rehabilitation. Admission type was determined by medical needs necessitating admission to inpatient (e.g., blood glucose monitoring in a patient with Type I diabetes), or insurance coverage. All participants underwent daily 1:1 physical therapy (PT) (2–3 h) and occupational therapy (OT) (2–3 h) 5 days per week. Inpatients also received 45 min of PT and 45 min of OT on each weekend day and public holiday. Functional activities included timed activities of stepping in and out of a tub, box and laundry carrying up and down stairs, total body movement including strength and cardiovascular activities, walking with a backpack, and desensitization for allodynia when appropriate. Patients received individual and group cognitive behavior therapy (CBT)-based interventions, provided by staff psychologists, in addition to coping support as needed during PT or OT sessions. Parents were invited to attend separate individual and parent group therapy sessions. Creative arts therapy was also incorporated into treatment, including weekly group and individual art therapy as well as weekly group music therapy. Duration of treatment was tailored to each individual patient’s needs, and typical length of stay in the program was 2–3 weeks. After discharge, patients were seen for a follow-up in approximately 2–3 months.

### 2.3. Data Collection and Methods

Quality improvement project data included pre- and post- program PedsQL SF-15 questionnaires completed by patient and a parent, as well as the family impact module completed by the parent. Data on pain and clinical characteristics abstracted from medical chart review included verbal pain intensity score (0–10), FDI (0–60; higher scores indicative of greater impact on a patient’s life), presence of allodynia, energy level (rated from 0–100%, with higher scores indicating greater energy level), presence of autonomic changes, and presence of somatic symptoms like nausea or fatigue. Past medical history and psychological history were abstracted from the medical record and included data collected via standardized intake surveys administered prior to the initial clinic visit and documented in the electronic health record, as well as data queried by the treating physician (approximately 90 min assessment) and/or the psychologist (approximately 60 min assessment) at the time of initial consultation. Furthermore, program participation information, such as the number of program participation days, any adverse events experienced during the program, and number of individual and group therapy sessions during the program were abstracted from retrospective chart review and input to a Research Electronic Data Capture (REDCap) database [12,13].

### 2.4. Clinical Characteristics and Demographics

Table 1 lists the outcome measures included in this study, including physical and psychosocial outcomes. Physical outcomes were based upon performance of therapy activities and the presence or intensity of a patient’s symptoms. PT and OT activity scores measured gross and fine motor function, balance, and coordination, including the Bruce treadmill test [14] and the Bruininks-Oseretsky Test of Motor Proficiency (BOT) [15]. The FDI score measured the perceived level of difficulty completing daily activities for the patient [16,17,18]. Patients self-reported their pain (on a scale of 0–10) and energy (on a scale of 0–100). The presence (or absence) of allodynia, adverse events, autonomic changes as defined by the Budapest criteria for complex regional pain syndrome [19], and somatic changes were abstracted from participants’ medical records into the REDCap database by the study team. The PedsQL-SF 15 Physical Module was also considered a physical outcome.

Psychosocial outcomes included the remaining modules of the PedsQL-SF 15 [9]: Emotional, Social, School, and Psychosocial. Also included in psychosocial outcomes was the PedsQL Family Impact Score [10], which consists of scales measuring parent-reported functioning in the following domains: Physical, Emotional, Social, Cognitive, Communication, and Worry, as well as parent-reported family functioning in Daily Activities and Family Relationships. These surveys were collected by members of the research team, not treatment team members, on RedCap to minimize both performance bias and measurement bias.

### 2.5. Study Objective

The primary study objective was to evaluate treatment outcomes pertaining to the HRQoL for both patients (PedsQL SF-15) and the family unit (PedsQL Family Impact module), before and after participation in the program.

### 2.6. Data Analyses

The total sample size for this study was based on a convenience sample of adolescents enrolled in the IIPT program over the study interval. Descriptive statistics on patient demographics and clinical characteristics were reported using the median and interquartile range (IQR) for continuous variables, and frequencies and percentages for categorical variables. Change in pain, clinical characteristics, patient and parent-reported PedsQL measure scores, parent-reported family impact scores, and PT and OT activities scores pre- and post- program participation were tested using Wilcoxon signed-rank test for continuous variables and McNemar test for categorical variables. We explored associations between changes in patient reported HRQoL pre- and post- program participation (dependent outcome variable), and all variables of interest (independent predictor variables) using simple and multiple linear regression models. Pairwise deletion was conducted to address missingness. Complete data analyses were conducted using SAS version 9.4 (Copyright© 2002–2012 by SAS Institute Inc., Cary, NC, USA).

## 3. Results

A total of 115 subjects participated in IIPT and completed the PedsQL questionnaire (58% of all patients treated in this time interval, total *n* = 199). As shown in Table 2, most patients were female (79%) and the median age at program entry was 15 (IQR: 12–16). The median number of program participation days was 17 (IQR: 14–19), with most patients being admitted to Day Hospital (87%), residing outside of Pennsylvania (53%), and presenting with history of at least one psychiatric diagnosis (73%). During the program participation, the median number of individual psychological therapy sessions received was 7 (IQR: 6–9) and the median number of parent group sessions (with at least one parent in attendance per patient) was 3 (IQR: 3–4).

Table 3 shows the changes in family impact scores using the pre- and post- program participation scores from the PedsQL family impact module. Overall, individual domains, as well as summary scores, were higher at discharge than at admission (Wilcoxon signed-rank test, all *p* < 0.01), suggesting improvement.

Changes in patient pain intensity, clinical characteristics, and quality of life were analyzed, with the results shown in Table 4. Patients’ verbal pain intensity score, FDI score [1], and self-perceived energy level improved at discharge (all *p* < 0.001). The presence of allodynia in patients was reduced at discharge, with 86% of patients reporting allodynia at admission and 61% at time of discharge (*p* < 0.001). The median patient PedsQL total score was 77 (IQR: 65–88) at discharge compared to 50 (IQR: 40–62; *p* < 0.001) at admission. All individual PedsQL domain scores, including physical functioning, social functioning, school functioning, emotional functioning, and psychosocial functioning, demonstrated statistically significant improvement at discharge when compared to admission scores (all *p* < 0.001).

Using bivariate linear regression, we explored factors associated with changes in patient-reported health-related quality of life (HRQoL) measured by the difference between PedsQL HRQoL scores pre- and post- program participation (Table 5). Improvement in patients’ verbal pain intensity score and FDI scores were associated with significant improvement in HRQoL. Specifically, with each point decrease in verbal pain intensity score at discharge, average patient-reported HRQoL score increased by approximately 2 points (β = 2.29, *p* < 0.01). Similarly, each point decrease in FDI score at discharge was associated with an average increase in HRQoL score by approximately 1 point (β = 0.96, *p* < 0.001). With each point increase in energy level at discharge, HRQoL scores improved by 0.19 (*p* < 0.001). Patients who experienced adverse events (AEs) during program participation reported HRQoL scores on average 11.24 points lower at discharge than those who did not experience any AEs. Improvement in total family impact score at discharge was associated with a 0.29 point increase in patient HRQoL at discharge (*p* < 0.01). Improvements in individual family impact domain levels, including emotional functioning, worry, daily activity, family function score, and parent HRQoL score were all associated with improvement in patient HRQoL score at discharge (all *p* < 0.05). In multivariable linear regression model, improvement in pain intensity score and FDI at discharge were independently associated with increased HRQoL scores at discharge, with β of 0.94 and 0.76, respectively (*p* < 0.05).

Table 6 highlights changes in pain and clinical characteristics upon discharge from the intensive program and at first follow-up. Comparing clinical characteristics pre- and post-participation revealed statistically significant improvements in pain intensity, self-reported daily functioning, and allodynia, all sustained at the first follow-up appointment (all *p* < 0.05). In contrast, while the presence of somatic symptoms, autonomic changes, and energy level significantly improved at program completion (all *p* < 0.01), the durability of these changes was not demonstrated among the study cohort at the first follow-up appointment (all *p* > 0.07).

Finally, Table 7 provides detailed information regarding the PT and OT activities evaluated in the program and reports outcomes demonstrated via treatment. Objective measures of physical function, strength, coordination, agility, and endurance all significantly improved after program completion (all *p* < 0.001).

## 4. Discussion

Overall, this study found that after participation in the IIPT, patients showed a decrease in symptoms of their idiopathic chronic pain disorder, allowing them to complete daily activities with less difficulty and experience less pain and hypersensitivity.

Treatment of pediatric chronic pain should follow a non-pharmacological IIPT approach including aerobic training, desensitization, and CBT, with a focus on helping patients decrease pain and pain-associated functional challenges and increase coping, while also providing psychoeducation and support for caregivers. Given the known impact of the family unit on the pain-related functioning and coping of youths with chronic pain, it is imperative to examine the effects of IIPT on both the level of the individual and the family unit. Following participation in a non-pharmacological IIPT program, patients demonstrated improvements in pain intensity as well as all domains of HRQoL. Parent-reported family impact scores from this time also demonstrated improvement for all HRQoL domains. Our study provides additional data to support the broad, positive impact of such a program, not only for the patient, but the family unit as well. Patients and their caregivers would benefit from additional education and counseling prior to enrollment in IIPT in order to align all stakeholders on treatment expectations, the patient’s recovery goals, and how the family unit can best offer support.

We found that, on average, pain intensity (0–10) was reduced from a median of 7 to 5 from program initiation to completion and was further reduced to a median of 2 by the first follow-up. Similarly, patients’ difficulty completing daily activities decreased significantly over study duration, with FDI scores at a mild to moderate range by the end of the study and remaining in that range at the time of first follow-up. Furthermore, allodynia significantly improved, and this effect was also sustained at the first follow-up appointment. It is noteworthy that somatic symptoms and fatigue did not demonstrate robust improvements, emphasizing the shared importance of lessening pain’s impact on the child’s social, family, and academic experiences, as well as their physical functioning, in alleviating their symptoms. This finding also underscores an ongoing need for services including psychology, psychiatry, and sleep hygiene, as well as additional workup to ensure that somatic changes are not due to a concurrent disease state external to a patient’s chronic pain diagnosis (e.g., a neurologic disorder).

Regression analyses suggested drivers of improved patient-level HRQoL upon program completion to be improvements in pain, self-reported functioning, and the overall HRQoL of the family unit. We hypothesize the HRQoL improvement seen at the family unit level may be due to the following factors: (1) it is distressing to see one’s child in pain, therefore as patients’ clinical status improved, parental distress and thereby HRQoL of the family unit improved; and (2) many parents received individual and parent group therapy sessions as additional psychological support throughout the treatment program. These sessions worked to redirect feelings of helplessness parents may experience while seeing their child in pain and focused instead on how parents could empower their child in their rehabilitation journey and support them when in distress. With a combination of patient- and parent-reported outcomes, this study shows the importance of targeting the entire family unit in intensive pain rehabilitation programs. We urge future researchers in this space to consider including family-level outcomes as an important component of treatment outcomes for pediatric chronic pain patients.

The study has several limitations. It is a retrospective study from a quality improvement project utilizing a convenience sample; thus, there is no placebo control group. That said, in the authors’ opinion, it would have been unethical to conduct a randomized placebo-controlled trial given the established efficacy of IIPT for pediatric chronic pain. Approximately 60% of patients in the program during the study timeframe were included. Inclusion was dependent on research team member and/or patient and family availability and administrators of the survey were blind to the treatment progress of patients, minimizing any bias. Since the source population for this IRB approved study was limited to those subjects who completed the PedsQL measures, we did not conduct secondary analyses to determine whether there were significant differences between those who participated in the study and those who did not, which may limit this study’s external validity. We did not examine race nor ethnicity on outcomes in this study. However, we are reassured that the patient demographics (sex and age) in this study were otherwise representative of our clinic population. Future work that is powered to assess whether there are any healthcare disparities based on racial and ethnic background is warranted. Additionally, parental involvement was limited to completing the PedsQL Family Impact module; parent engagement with therapy sessions was not collected or compared to any changes in their survey data.

Another limitation to consider is that patients admitted to the IIPT generally have more limitation in function due to their pain and therefore may not represent a generalizable population of youth with idiopathic chronic pain. To be eligible for program entry, participants needed to have (1) stable, if any, psychiatric conditions (including no active eating disorders), (2) demonstrated readiness for participation in an intensive pain rehabilitation program, and (3) no active co-morbid medical diagnoses necessitating additional diagnostic evaluation or initiation of new medications. Another limitation was that behavioral health diagnoses were limited to self-report rather than formal assessment. Patients with diagnoses of intellectual or developmental disabilities, despite their increased likelihood of chronic pain, [20], were not included in this study. This exclusion was not a purposeful choice by investigators but perhaps a testament to the increased difficulty of detecting pain in individuals with communication-related disabilities, [20] or this population’s challenges accessing the healthcare system.

Other limitations include the lack of assessment regarding the economic cost of the program; however, this has recently been demonstrated in another study [21]. “Improving HRQoL” is an inherently limited shorthand for the metrics shown in this paper: decrease in pain and other unpleasant symptoms, increase in ability to complete desired daily tasks at home and school, and increase in positive relationships within the family unit. Perhaps a simplification of a more nuanced concept, this study in no way diminishes the lives of those who score lower on these metrics, but rather emphasizes additional advancements needed to more fully serve all patients. Our study would have been strengthened by a repeat assessment of HRQoL at the time of the first follow-up after discharge from the program, as well as patient assessment beyond 3 months after program completion. Durability of these changes in HRQoL scores would be an important focus for future longitudinal, prospective studies in this patient population, and additional work is warranted to identify any patient characteristics that make a child more likely to exhibit durable program results, such as lower initial pain scores, length of time in the program, etc.

## 5. Conclusions

A nonpharmacologic IIPT is a compelling treatment for adolescents with chronic idiopathic pain, benefiting both patients and the family unit. The study results support the non-pharmacological interdisciplinary approach as evidence-based treatment of pediatric chronic pain.

## Figures and Tables

**Table 1:** Study Outcome Measures.

**A. Physical Outcomes**	
Measure	Description
Occupational and physical therapy activity scores	Timed activities (tub step in’s, box carry, reverse box carry, shoulder carry, laundry carry) BOT scores [15], percentile and standard score, which measures gross motor function, fine motor function, balance, and coordination (fine manual control, manual coordination, body coordination, strength and agility). Bruce treadmill test [14]—a participant is walking or running on a treadmill, with increasing speed and incline, every 3 min, until they are no longer able to or at end of test time of 21 min.
FDI (Functional Disability Inventory) score	Total score 0–60 [16,18] based on level of difficulty in performing daily activities in different settings (home, school, social, recreational); higher scores indicate greater difficulty functioning with scores categorized as follows: no/minimal (0–12), mild (13–20), moderate (21–29) and severe (≥30) disability.
Verbal pain intensity score	Self-reported level of pain 0–10 scale (0 = no pain, 10 = unbearable pain)
Energy level	Self-perceived level of energy 0–100 scale (0 = no energy, 100 = extremely high energy)
Allodynia	Presence—yes or no of pain with a typically non-painful stimulus
Adverse event	Presence of adverse event at end of study (e.g., physical injury, early discharge due to psychological distress)
Autonomic change	Presence of autonomic symptoms (as per the Budapest Criteria for Complex Regional Pain Syndrome) [19]
Somatic change	Presence of somatic symptoms (e.g., nausea, headaches)
Pediatric Quality of Life inventory short form (PedsQL SF-15)—Physical module [9]	The patient and parent report 15-item PedsQL measure includes 5 modules measuring: (1) Physical Functioning (2) Emotional Functioning (3) Social Functioning (4) School Functioning and (5) Psychosocial Functioning. Only module 1 is included in the physical outcomes of this paper.
**B. Psychosocial Outcomes**	
Measure	Description
Pediatric Quality of Life inventory (PedsQL) Family impact score [10]	The 36-item PedsQL Family Impact Module Scales encompass 6 scales measuring Parent Self-Reported Functioning: (1) Physical Functioning (6 items), (2) Emotional Functioning (5 items), (3) Social Functioning (4 items), (4) Cognitive Functioning (5 items), (5) Communication (3 items), (6) Worry (5 items), and 2 scales measuring Parent-Reported Family Functioning; (7) Daily Activities (3 items) and (8) Family Relationships (5 items). A 5-point response scale is utilized (0 = never a problem; 4 = always a problem). Items are reverse-scored and linearly transformed to a 0–100 scale (0 = 100, 1 = 75, 2 = 50, 3 = 25, 4 = 0), so that higher scores indicate better functioning (less negative impact).
Pediatric Quality of Life inventory short form (PedsQL SF-15) [9]	The patient-reported 15-item PedsQL measure includes 5 modules measuring: (1) Physical Functioning (2) Emotional Functioning (3) Social Functioning (4) School Functioning and (5) Psychosocial Functioning. Modules 2, 3, 4, and 5 are included in the psychosocial outcomes of this paper.

**Table 2:** Demographics and Patient Characteristics (*n* = 115).

Variables	Value, *n* (%) or Median (IQR)
Age at admission,	15 (12–16)
Sex, female	91 (79%)
State of residence, Pennsylvania	54 (47%)
Day hospital admission	100 (87%)
Number of days participated in the program	17 (14–19)
History of mental health condition—Patient ^†^	84 (73%)
Major depressive disorder	2 (2%)
Unspecified depression	46 (40%)
Generalized anxiety disorder	7 (6%)
Unspecified anxiety disorder	57 (50%)
Post-traumatic stress disorder	4 (3%)
Patient on at least one psychiatric medication	49 (43%)
During Treatment Program	
Number of individual counseling sessions	7 (6–9)
Number group counseling sessions	3 (3–4)
Number of group parent counseling sessions	3 (3–4)
Adverse event ^†^	
Early discharge	4 (3%)
Infection	1 (1%)
Injury/trauma	5 (4%)
Patient/family choice	1 (1%)
Other	5 (4%)
At least one adverse event	15 (13%)
Attempted coordination of care with outpatient psychologist	86 (75%)
Time to 1st follow-up post discharge (days)	73 (59–89)

^†^ Patients could present with more than one of these items (e.g., more than one mental health condition, more than one adverse event).

**Table 3:** Change in Pediatric Quality of Life Family Impact Score (*n* = 113) *.

Domains	Score at Admit	Score at Discharge	Direction of Change	*p*-Value
Physical functioning	58 (46–71)	67 (58–75)	↑	<0.0001
Emotional functioning	55 (45–65)	70 (55–80)	↑	<0.0001
Social functioning	69 (50–81)	75 (63–94)	↑	<0.0001
Cognitive functioning	65 (50–80)	75 (55–90)	↑	<0.01
Communication	58 (42–75)	67 (58–83)	↑	<0.0001
Worry	50 (35–60)	65 (50–75)	↑	<0.0001
Daily activities	50 (42–67)	67 (50–83)	↑	<0.0001
Family relationships	65 (50–75)	70 (55–80)	↑	<0.0001
**Summary Scores**				
Parent HRQoL	60 (48–73)	71 (59–80)	↑	<0.0001
Family functioning	59 (47–72)	69 (56–81)	↑	<0.0001
Total family score	58 (45–67)	70 (58–79)	↑	<0.0001

* 2 subjects were removed from the analysis due to missing discharge visit data on PedsQL Family Impact Measures. *p*-value < 0.05 considered statistically significant; The 36-item PedsQL Family Impact Module Scales encompass 6 scales measuring parent self-reported functioning: (1) Physical Functioning (6 items), (2) Emotional Functioning (5 items), (3) Social Functioning (4 items), (4) Cognitive Functioning (5 items), (5) Communication (3 items), (6) Worry (5 items), and 2 scales measuring parent-reported family functioning; (7) Daily Activities (3 items) and (8) Family Relationships (5 items). A 5-point response scale is utilized (0 = never a problem; 4 = always a problem). Items are reverse-scored and linearly transformed to a 0–100 scale (0 = 100, 1 = 75, 2 = 50, 3 = 25, 4 = 0), so that higher scores indicate better functioning (less negative impact). Direction of change indicated by “↑” signifies a higher value for the respective variable at time of discharge.

**Table 4:** Change in Pain, Clinical Characteristics and Quality of Life (*n* = 115).

Variables	Score at Admit	Score at Discharge	Direction of Change	*p*-Value
Verbal pain intensity score (0–10)	7 (6–9)	5 (3–8)	↓	<0.0001
FDI (0–60)	26 (17–34)	9 (4–15)	↓	<0.0001
Allodynia, yes	98 (86%)	70 (61%)	↓	<0.0001
Energy level	70 (50–80)	77 (60–90)	↑	<0.001
**Pediatric Quality of Life Short Form Patient Measures**				
Physical functioning	30 (15–45)	80 (60–90)	↑	<0.0001
Emotional functioning	56 (38–75)	75 (63–94)	↑	<0.0001
Social functioning	92 (75–100)	100 (75–100)	↑	<0.0001
School functioning	50 (25–75)	67 (50–83)	↑	<0.0001
Psychosocial functioning	60 (48–73)	80 (68–90)	↑	<0.0001
Total PedsQL score	50 (40–62)	77 (65–88)	↑	<0.0001

*p*-value < 0.05 considered statistically significant. Missing data: FDI at admit (*n* = 114), energy at admit (*n* = 110), Verbal pain at discharge (*n* = 114), energy at discharge (*n* = 111), FDI at discharge (*n* = 112), Allodynia (*n* = 114); Continuous variable tested using Wilcoxon Signed-Rank test, categorical variable tested using McNemar Test; MCID (minimal clinically important difference) for verbal pain intensity = 3 and FDI = 8. Arrows indicate either an increase (↓) or decrease (↓) of the respective variable at time of discharge.

**Table 5:** Factors Associated with Change in Patient Reported Health-Related Quality of Life (HRQoL) (*n* = 115).

Variables	Unadjusted β	95% CI		*p*-Value	Adjusted β	95% CI		*p*-Value
Adverse event, yes	−11.24	−19.46	−3.02	<0.01	−2.65	−10.44	5.15	0.50
∆ Verbal pain intensity score	2.29	1.33	3.24	<0.0001	0.94	0.05	1.82	0.04
∆ FDI	0.96	0.76	1.15	<0.0001	0.76	0.53	1.00	<0.01
∆ Energy	0.19	0.07	0.31	<0.01	−0.004	−0.11	0.10	0.94
∆ Family impact score	0.29	0.10	0.48	<0.01	0.13	−0.02	0.29	0.09
∆ Emotional functioning	0.17	0.02	0.31	0.03	-	-	-	-
∆ Worry	0.22	0.09	0.35	<0.01	-	-	-	-
∆ Daily activity score	0.19	0.08	0.31	<0.001	-	-	-	-
∆ Family function score	0.22	0.05	0.39	<0.01	-	-	-	-
∆ Parent HRQoL score	0.20	0.03	0.37	0.02	-	-	-	-

*p*-value < 0.05 considered statistically significant. ∆ = Change in score; Where, ∆ verbal pain intensity score = score at admission − score at discharge, ∆ Functional Disability Inventory (FDI) = score at admission − score at discharge, ∆ Energy = score at discharge − score at admission, ∆ Family Impact Scores (total and individual domains) = score at discharge − score at admission.

**Table 6:** Changes in Pain and Clinical Characteristics at Completion of Treatment and First Follow-up (*n* = 115).

Variables	Score at Admit	Score at Discharge	Score at 1st Follow-Up	*p*-Value	
				Program Entry vs. End	Program Completion vs. 1st Follow-Up
Verbal pain intensity score (range: 0–10)	7 (6–9)	5 (3–8)	2 (0–5)	<0.0001	<0.0001
FDI (range: 0–60)	26 (17–34)	9 (4–15)	6 (1–13)	<0.0001	0.04
Allodynia, yes	98 (86%)	70 (61%)	32 (33%)	<0.0001	<0.0001
Energy level (range: 0–100)	70 (50–80)	77 (60–90)	80 (60–90)	<0.001	0.07
Autonomic change, yes	10 (9%)	2 (2%)	3 (3%)	<0.01	0.56
Somatic change, yes	102 (89%)	22 (19%)	31 (33%)	<0.001	0.17

*p*-value < 0.05 considered statistically significant. Admission: Functional Disability Inventory (FDI) (*n* = 114), energy (*n* = 110), Discharge: Verbal pain(*n* = 114), energy (*n* = 111), FDI (*n* = 112), Allodynia (*n* = 114); Follow-up: Verbal pain (*n* = 96), FDI (*n* = 95), energy (*n* = 93), Allodynia (*n* = 96), Autonomic change (*n* = 96); Continuous variable tested using Wilcoxon Signed-Rank test, categorical variable tested using McNemar Test.

**Table 7:** Changes in PT and OT Activity Scores (*n* = 115).

	Score at Admit	Score at Discharge	Direction of Change	*p*-Value
Bruce treadmill test	11 (8–13)	15 (13–16)	↑	<0.0001
Timed activities:				
Box carry (seconds)	67 (56–93)	26 (24–29)	↓	<0.0001
Reverse box carry (seconds)	67 (55–99)	27 (25–30)	↓	<0.0001
Shoulder carry (seconds)	39 (30–54)	13 (12–14)	↓	<0.0001
Laundry carry (seconds)	76 (60–103)	42 (38–49)	↓	<0.0001
Tub step-ins (number completed in 60 s)	20 (15–23)	34 (32–35)	↑	<0.0001
BOT Scores:				
Fine manual control (percentile)	35 (21–54)	66 (42–89)	↑	<0.0001
Fine manual control (standard score)	46 (42–51)	54 (48–62)	↑	<0.0001
Manual coordination (percentile)	38 (14–58)	73 (54–93)	↑	<0.0001
Manual coordination (standard score)	47 (39–52)	56 (51–65)	↑	<0.0001
Body coordination (percentile)	18 (6–52)	58 (26–76)	↑	<0.0001
Body coordination (standard score)	41 (34–50)	52 (44–57)	↑	<0.0001
Strength and agility (percentile)	17 (6–50)	58 (27–79)	↑	<0.0001
Strength and agility (standard score)	41 (34–50)	52 (45–58)	↑	<0.0001

Significant *p*-value; Missing Data admission visit: Tub steps in (4), box carry (3), reverse box carry (7), shoulder carry (7), laundry carry (3), fine manual control percentile (1), fine manual control standard (2), manual coordination percentile (2), manual coordination standard (3), body coordination percentile (7), body coordination standard (10), strength and agility percentile (7), strength and agility standard (10), Bruce treadmill test (2); Missing Data discharge visit: Tub steps in (5), box carry (3), reverse box carry (7), shoulder carry (7), laundry carry (3), fine manual control percentile (5), fine manual control standard (8), manual coordination percentile (5), manual coordination standard (8), body coordination percentile (7), body coordination standard (10), strength and agility percentile (8), strength and agility standard (11), Bruce treadmill test (6).

## Data Availability

The data presented in this study are available on request from the corresponding author. The data are not publicly available due to privacy and ethical reasons as well as requirements from the Institutional Review Board of The Children’s Hospital of Philadelphia.

## Abbreviations

HRQoL = health-related quality of life, IIPT = intensive interdisciplinary pain treatment, Pediatric Quality of Life Inventory Short Form 15 = PedsQL SF-15, PT = physical therapy, OT = occupational therapy, CBT = cognitive behavioral therapy, FDI = Functional Disability Inventory, BOT = Bruininks-Oseretsky Test of Motor Performance.

## References

[1] Hassett A.L., Hilliard P.E., Goesling J., Clauw D.J., Harte S.E., Brummett C.M. (2013). Reports of Chronic Pain in Childhood and Adolescence among Patients at a Tertiary Care Pain Clinic. J. Pain.

[2] Groenewald C.B., Law E.F., Fisher E., Beals-Erickson S.E., Palermo T.M. (2019). Associations Between Adolescent Chronic Pain and Prescription Opioid Misuse in Adulthood. J. Pain.

[3] Harrison L.E., Pate J.W., Richardson P.A., Ickmans K., Wicksell R.K., Simons L.E. (2019). Best-Evidence for the Rehabilitation of Chronic Pain Part 1: Pediatric Pain. J. Clin. Med..

[4] Hoffart C., Wallace D. (2014). Amplified pain syndromes in children: Treatment and new insights into disease pathogenesis. Curr. Opin. Rheumatol..

[5] Feldman D.E., Nahin R.L. (2021). National Estimates of Chronic Musculoskeletal Pain and Its Treatment in Children, Adolescents, and Young Adults in the United States: Data from the 2007–2015 National Ambulatory Medical Care Survey. J. Pediatr..

[6] Sherry D.D., Brake L., Tress J.L., Sherker J., Fash K., Ferry K., Weiss P.F. (2015). The Treatment of Juvenile Fibromyalgia with an Intensive Physical and Psychosocial Program. J. Pediatr..

[7] Fisher E., Villanueva G.M., Henschke N., Nevitt S.J., Zempsky W.M., Probyn K., Buckley B., Cooper T.E.M., Sethna N.M., Eccleston C. (2022). Efficacy and safety of pharmacological, physical, and psychological interventions for the management of chronic pain in children: A WHO systematic review and meta-analysis. Pain.

[8] Palermo T.M., Walco G.A., Paladhi U.R., Birnie K.A., Crombez G., de la Vega R., Eccleston C., Kashikar-Zuck S., Stone A.L. (2021). Core outcome set for pediatric chronic pain clinical trials: Results from a Delphi poll and consensus meeting. Pain.

[9] Varni J.W., Burwinkle T.M., Seid M. (2005). The PedsQL™ as a pediatric patient-reported outcome: Reliability and validity of the PedsQL™ Measurement Model in 25,000 children. Expert Rev. Pharmacoecon. Outcomes Res..

[10] Varni J.W., Sherman S.A., Burwinkle T.M., Dickinson P.E., Dixon P. (2004). The PedsQL family impact module: Preliminary reliability and validity. Health Qual. Life Outcomes.

[11] Desai A.D., Zhou C., Stanford S., Haaland W., Varni J.W., Mangione-Smith R.M. (2014). Validity and responsiveness of the pediatric quality of life inventory (PedsQL) 4.0 generic core scales in the pediatric inpatient setting. JAMA Pediatr..

[12] Harris P.A., Taylor R., Minor B.L., Elliott V., Fernandez M., O’Neal L., McLeod L., Delacqua G., Delacqua F., Kirby J. (2019). The REDCap consortium: Building an international community of software platform partners. J. Biomed. Inform..

[13] Harris P.A., Taylor R., Thielke R., Payne J., Gonzalez N., Conde J.G. (2009). Research electronic data capture (REDCap)—A metadata-driven methodology and workflow process for providing translational research informatics support. J. Biomed. Inform..

[14] Gumming G.R., Everatt D., Hastman L. (1978). Bruce treadmill test in children: Normal values in a clinic population. Am. J. Cardiol..

[15] Deitz J., Kartin D., Kopp K. (2007). Review of the Bruininks-Oseretsky Test of Motor Proficiency, Second Edition (BOT-2). Phys. Occup. Ther. Pediatr..

[16] Kashikar-Zuck S., Flowers S.R., Claar R.L., Guite J.W., Logan D.E., Lynch-Jordan A.M., Palermo T.M. (2011). Clinical utility and validity of the Functional Disability Inventory among a multicenter sample of youth with chronic pain. Pain.

[17] Flowers S.R., Kashikar-Zuck S. (2011). Measures of juvenile fibromyalgia: Functional Disability Inventory (FDI), Modified Fibromyalgia Impact Questionnaire–Child Version (MFIQ-C), and Pediatric Quality of Life Inventory (PedsQL) 3.0 Rheumatology Module Pain and Hurt Scale. Arthritis Care Res..

[18] Walker L.S., Greene J.W. (1991). The Functional Disability Inventory: Measuring a Neglected Dimension of Child Health Status. J. Pediatr. Psychol..

[19] Harden R.N., Bruehl S., Perez R.S.G.M., Birklein F., Marinus J., Maihofner C., Lubenow T., Buvanendran A., Mackey S., Graciosa J. (2010). Validation of proposed diagnostic criteria (the “Budapest Criteria”) for Complex Regional Pain Syndrome. Pain.

[20] Breau L.M., Burkitt C. (2009). Assessing pain in children with intellectual disabilities. Pain Res. Manag..

[21] Lopez Lumbi S., Ruhe A., Pfenning I., Wager J., Zernikow B. (2021). Economic long-term effects of intensive interdisciplinary pain treatment in paediatric patients with severe chronic pain: Analysis of claims data. Eur. J. Pain.

